# Do sex differences in reported weight loss intentions and behaviours persist across demographic characteristics and weight status in youth? A systematic review

**DOI:** 10.1186/s12889-018-6179-x

**Published:** 2018-12-04

**Authors:** Stephanie A. Houle-Johnson, Lisa Kakinami

**Affiliations:** 1 Department of Psychology, Concordia University, Montreal, Quebec Canada; 2 School of Psychology, University of Ottawa, 136 Jean-Jacques Lussier Private, VNR3088, Ottawa, ON K1N 9A8 Canada; 3 Department of Mathematics and Statistics, Concordia University, 1455 De Maisonneuve Blvd. W., Room S-LB 927, Montreal, QC H3G 1M8 Canada; 4 PERFORM Centre, Concordia University, Montreal, Quebec, Canada

**Keywords:** Weight loss, Obesity, Systematic review, Youth

## Abstract

**Background:**

Evidence suggests that young females are more likely to try to lose weight than young males, however whether this sex difference persists across demographic characteristics and weight status is unclear. Further, whether females are more likely than males to try to lose weight using unhealthy weight loss strategies has never been systematically assessed. The objective of this systematic review was to examine the literature on sex differences in weight loss intentions and strategies in children and adolescent observational studies to determine whether sex differences persisted across demographic characteristics (race/ethnicity, grade level) and weight status.

**Methods:**

Relevant articles published after 1990 were identified using PubMED, Web of Science, and PsycInfo. Searches were conducted in May of 2015 and again in May of 2017. Studies conducted in the US and Canada with participants 18-years old or younger who measured weight loss strategies in the context of weight loss intention were selected. Descriptive statistics were extracted from 19 studies.

**Results:**

Almost two-thirds of youth reported trying to lose weight. High-school and middle-school aged females reported consistently higher prevalence of weight loss intentions compared to male counterparts, as did Caucasian, African-American, and Hispanic females. The proportion of youth using unhealthy or extreme strategies reached 44 and 13%, respectively, with a similar proportion of males and females endorsing the use of each category of weight loss strategies across studies. Native-American youth reported the highest prevalence (27%) of using extreme strategies.

**Conclusions:**

Researchers should consider demographic characteristics when reporting prevalence information for weight loss intentions and behaviours, as certain groups might require more targeted public health initiatives. Across characteristics, prevalence ranges were broad for weight loss intentions and use of particular strategies, suggesting the need to standardize and refine data collection and reporting practices in this literature.

**Electronic supplementary material:**

The online version of this article (10.1186/s12889-018-6179-x) contains supplementary material, which is available to authorized users.

## Background

Approximately one-third to one-half of high school students report trying to lose weight [1, 2]. Nearly half will either fail to lose weight, or will regain the weight [3–6]. While a combination of dietary changes and increased physical activity is recommended [7], considerable heterogeneity in weight loss strategies have been reported [1]. Importantly, the effectiveness of these behaviours on weight loss differs between strategies, and some strategies may not only be ineffective, but may also be counterproductive [8–10]. For instance, Neumark-Sztainer et al. [9] reported greater increases in body mass index (BMI) over 10-years of follow-up among adolescents who reported unhealthy weight loss behaviours (such as fasting, using laxatives, etc.) compared to those who didn’t report unhealthy weight loss behaviours [9]. Another adverse consequence of unhealthy weight loss strategies include the potential development of eating disorders, which has important implications for health later in life [11–14].

As weight loss intentions and weight loss strategies tend to track from adolescence into adulthood [9], improving our understanding of how youth populations self-manage weight loss is critical for curbing the obesity epidemic. Indeed, both weight loss intentions and the use of particular weight loss strategies reportedly differs based on demographic and health characteristics such as age [12], race/ethnicity [15], or weight status [16, 17]. For instance, while studies have demonstrated that females are more likely than males to try to lose weight [18–21] and to do so using unhealthy behaviours [15, 22, 23], findings as to whether this sex difference persists in weight loss intentions and strategies across other demographic and health characteristics have been mixed. While some studies report significant sex differences across age [24], others report no sex differences in demographic sub-groups (e.g., Native-American youth) [20, 21, 25]. Understanding where sex differences persist in the prevalence of using healthy and unhealthy weight loss behaviours among these different demographic groups would allow for the development and execution of more refined public health initiatives aimed at reducing obesity in youth. Indeed, research suggests that targeted health initiatives can add benefits that universal approaches to public health messaging cannot provide [26, 27].

No systematic review has yet examined sex differences within demographic characteristics or weight status in the context of weight loss intention in observational studies conducted among children and adolescents. Previous reviews have focused on clinical trials [28–31], and whether a sex difference persists in observational studies is largely unknown. Discerning whether a sex difference in weight loss intentions and strategies persists across other demographic characteristics and weight status will improve researchers’ and clinicians’ ability to identify subpopulations of youth at the highest risk for unhealthy weight loss behaviours, thus supporting efforts aimed at preventing obesity and other problematic health conditions related to weight mismanagement. The objective of this paper was to systematically review the literature on weight loss intention and strategies among children and adolescents from observational studies, and to estimate whether sex differences in these outcomes may persist across age, race/ethnicity and weight status.

## Method

### Search strategy

**Fig. 1:**
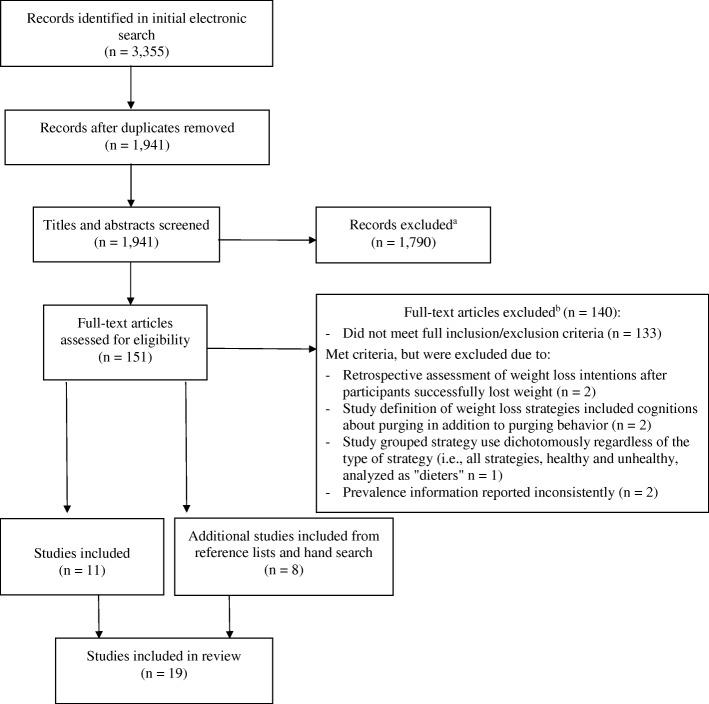
Review strategy decision tree. ^a^Records were excluded by screening titles and articles for relevance against inclusion and exclusion criteria. ^b^In addition to those studies that did not meet the inclusion and exclusion criteria, details of studies excluded based on quality or lack of fit with the review question are shown here

Existing literature published between January 1990 and May 2017 was searched electronically using three different databases: PubMED, Web of Science, and PsycInfo. The full search strategy is available in Additional file 1. The first search was conducted in May of 2015, during which 3355 articles were identified (Fig. 1). A second search was conducted in May of 2017 and yielded no new results.

### Eligibility criteria

The criteria for inclusion in the review were: (1) participants were 18 years of age or younger, (2) past or current weight loss intention alongside use of weight loss strategies were measured (i.e., either a subsample of those reporting weight loss intentions was analyzed, or question wording was such that weight loss intention could be assumed, such as “do you exercise to lose weight”), (3) the study used a Canadian or American sample, (4) the article was written in English, and (5) the study reported data on at least one of the characteristics of interest to the review. Articles were excluded if: (1) the study involved an intervention (e.g., behavioural counseling for weight loss with no baseline data on behaviours provided), (2) the study sample was an exclusively at-risk or other special population (e.g., diagnosed with an eating disorder), (3) weight loss strategies were too broadly defined without detail (e.g., “Do you diet to lose weight?”), or (4) the study was qualitative in nature. Of note, studies that assessed weight intention by combining weight loss and weight maintenance objectives (e.g., “do you exercise to lose weight or keep from gaining weight”) were excluded, as research suggests that weight loss and weight maintenance are cognitively different objectives that may lead to different health outcomes [32].

This review focuses on the United States and Canada, given that weight loss intentions are notably highest among this population of youth [33], more data is available for this region than in other countries, and the makeup of characteristics of interest to this review (e.g., race/ethnicity) likely differ substantially in other regions. Further, as the intent of this research is to highlight areas where further research is needed to better inform decisions made by public health agencies, relative homogeneity amidst study samples should allow for more generalizable findings. Studies excluded from other countries include those conducted in Australia and New Zealand, Eastern Europe, China, Iran, India, Ireland, Mauritius, Spain, Sweden, Switzerland, and Taiwan.

### Study selection

All records identified in the search were retrieved. The software EndNote X7 [34] was used to filter duplicate records and organize references. Both authors independently reviewed the titles and abstracts for articles considered for inclusion. Disagreements were resolved through discussion. Reference lists of studies deemed relevant for the review were examined to identify additional studies for inclusion. The reference lists of those identified were then consulted, and so on, until no additional articles could be identified as relevant for inclusion. During this process, 51 additional articles met the necessary criteria, and were deemed relevant for consideration in the review.

### Data extraction

Descriptive data across studies on weight loss intentions and strategies were extracted in order to report overall prevalence, and prevalence according to the following sub-categories: (1) weight status (underweight, normal-weight, and overweight/obese), (2) grade level (elementary school [approximately ages 5–10], middle school [ages 11–13], and high school [ages 14–18]), and (3) race/ethnicity (African American, Asian American, Caucasian, Hispanic, Native American). Additional details on the coding of these sub-categories are provided in their relevant results sections. Sex was taken into consideration within each of these subsections. Both authors were involved in the data extraction process – the first author conducted the primary data extraction, which was then verified by the second author. Weight loss strategies were broadly grouped into three categories based on those proposed by Neumark-Sztainer and colleagues (healthy, unhealthy, and extreme; see Table 1) [35]. In general, healthy behaviours are those that are recommended for weight management (e.g., exercise, dietary changes), while unhealthy and extreme behaviours (e.g., laxative use, fasting) are those that do not provide adequate nutrient intake, with extreme behaviours representing those that are most dangerous and counterproductive to health [35, 36].

**Table 1:** Studies examining weight loss intention and strategies in youth observational studies (*N* = 19)

Study	*N*	Sample^✝^	Gender (% female)	% OW/OB^a^	Ethnicity^✝^	Location	Study type	Healthy strategies	Unhealthy strategies	Extreme strategies
Calderon et al. (2004) [18]	146	10th grade	64	27 (M^b^)	8% Caucasian 27% Asian-American 4% African-American 48% Hispanic 0% Native-American 12% Other	California	CS^c^	Eating smaller portions	Skipping meals, fad or crash diet	Diet aids
Childress et al. (1993) [24]	3129	5th to 8th grade	51	N/A	64% Caucasian 21% African-American 7% Other 8% N/A	South Carolina	CS	Exercise	Fasting	Vomiting, diuretics, diet pills, laxatives
Davis & Lambert (2000) [25]	1994	5th grade	51	34 (M)	100% Native-American	New Mexico	CS	Exercise, “eating a little less”	Strict diet	N/A
French et al. (1995) [46]	852	7th to 10th grade	100	N/A	89% Caucasian 4% Mixed Race 3%Asian-American < 1%Native-American < 1%African-American < 1% Hispanic	Minnesota	L^d^	Low-calorie diet	N/A	Vomiting, diet pills, laxatives, enemas
Kilpatrick et al. (1999) [19]	6504	7th to 12th grade	53	22 (M) 31 (P^e^)	62% Caucasian 23% African-American 9% Hispanic 3% Asian-American 3% Native-American	USA^†^	CS	Exercise	N/A	Vomiting, diet pills, laxatives
Koff & Rierdan (1991) [45]	206	6th grade	100	21 (P)	100% Caucasian	Massachusetts	CS	Exercise, avoid fattening foods	Skipping meals, fasting	N/A
Krowchuk et al. (1998) [23]	2331	6th to 8th grade	49	24 (P)	64% Caucasian 28% African-American 2% Hispanic 1% Asian-American 2% Native-American 3% Other	North Carolina	CS	Exercise	N/A	Vomiting or using laxatives, diet pills
McVey et al. (2005) [20]	1458	6th to 8th grade	54	19 (M) 28 (P)	38% Caucasian 26% South Asian 17% Asian-American 13% African-Canadian 14% Other	Ontario, CA	CS	Exercise	Skipping meals	Vomiting, diet pills, laxatives or diuretics
Page et al. (1993) [49]	1915	10th to 12th grade	51	N/A	73% Caucasian 25% African-American 2% Other	Mississippi	CS	Exercise	Fasting, very restrictive diet, crash diet	Vomiting, diet pills, water pills, laxatives
Phelps et al. (1993) [40]	367^f^	7th to 8th grade and 9th to 12th grade	100	N/A	N/A	New York	CS	N/A	N/A	Vomiting, diet pills, laxatives
Rafiroiu et al. (2000) [43]	1439	3rd to 5th grade	52	N/A	55% Caucasian	South Carolina	CS	N/A	N/A	Vomiting, diet pills
Serdula et al. (1993) [39]	11,467	9th to 12th grade	51	25 (P)	55% Caucasian 20% African-American 20% Hispanic 6% Other	USA^‡^	CS	Exercise	Skipping meals	Vomiting, diet pills
Shisslak et al. (1998) [48]	523	Elementary and middle school children	100	N/A	48% Caucasian 26% Hispanic 17% Asian-American 5% African-American 2% Native-American 2% Other	California, Arizona	CS	Exercise, eat less fat/sweets	Skipping meals, fasting	Vomiting, diet pills, laxatives
Shisslak et al. (2006) [44]	1164	6th to 9th grade	100	19% overweight, 19% at risk overweight (M)	43% Caucasian 40% Hispanic 17% African-American	California, Arizona	CS	Exercise, cut back on food, eaten less sweets or fatty foods	Skipping meals, fasting	Vomiting, diet pills, laxatives
Stevens et al. (1999) [41]	304	4th grade	54	12 (P)	100% Native-American	Arizona, New Mexico, South Dakota	CS	Exercise, change what/how ate	Skipping meals, fasting	N/A
Story et al. (1994) [47]	13,454	7th to 12th grade	N/A	31 (P)	100% Native-American	South Dakota, New Mexico, Alaska, Minnesota, Montana, Tennessee, Utah, Arizona	CS	N/A	N/A	Vomiting, diet pills, laxatives, diuretics
Story et al. (2001) [21]	1441	2nd to 3rd grade	48	42 (M)	100% Native-American	Arizona, New Mexico, South Dakota	CS	Exercise, changed what/how much ate	Skipping meals, fasting	N/A
Yost et al. (2010) [38]	1040	Females aged 13–18	100	15.5% overweight, 11.8% obese (as per BMI percentile)	69% Caucasian 19% African-American 2% Native-American 3% Asian-American 12% Hispanic	USA^††^	CS	Exercise	N/A	N/A
Zullig et al. (2006) [42]	4175	9th to 12th grade	54	54 (P)	54% Caucasian 46% African-American	South Carolina	CS	N/A	Fasting	Vomiting or laxatives, diet pills

Note: ^✝^Sample reported here in terms found in the original article. Ethnicity reported here in the order found in the original article. ^a^*OW/OB* overweight/obese, ^b^*M* Measured OV/OB, ^c^*CS* Cross-sectional, ^d^*L* Longitudinal; ^e^*P* self-perception OV/OB, ^f^In this study, students were assessed at three time points (1984, 1989 and 1992). Results from the most recent wave of the study (1992) are presented in this review. ^†^Nationally representative sample: Add Health Wave I; ^††^Nationally representative sample: Add Health Wave I and II; ^‡^Nationally representative sample: Youth Risk Behavior Survey

### Quality and risk of Bias assessments

Both authors independently assessed the quality (pertaining to all aspects of the publication including introduction, methods, results and discussion) and potential bias of each study using the AXIS tool. Disagreements were resolved through discussion. The AXIS tool was specifically designed to be utilized across disciplines to critically appraise cross-sectional studies. With an international panel of 18 experts, the AXIS tool was developed through several iterative rounds of a Delphi process in order to reach consensus [37].

## Results

Based on titles and abstracts, 151 articles from peer-reviewed journals containing the variables of interest were deemed relevant for consideration in the review (Fig. 1). After applying the inclusion and exclusion criteria (see Fig. 1), 19 studies were retained for the final review.

### Study characteristics: Full sample

The demographic characteristics of each study sample are presented (Table 1). All but one study [20] was conducted in the United States; three used data from large, nationally representative datasets (Add Health [19, 38], and the Youth Risk Behavior Survey) [39]. Eighteen of the nineteen studies were cross-sectional. For the one longitudinal study, only data from the last year of study is reported here [40].

Exercise was the most commonly assessed healthy weight loss strategy (13/19 studies), while the prevalence of using dietary changes was the least frequently assessed (8/19 studies). Over half the studies assessed the use of unhealthy strategies (12/19), and three-quarters assessed the use of extreme strategies (14/19 studies). Skipping meals and fasting were the most commonly assessed unhealthy strategies (8/19) and using diet pills/aids was the most commonly assessed extreme strategy (14/19). No studies evaluated the use of multiple strategies simultaneously.

### Quality and risk of Bias assessments

Results of the quality assessment are shown (Table 2). None of the studies provided a power calculation or sample size justification. Non-response bias was not a concern for seven studies, while sampling procedures from two studies raised concern about non-response bias. There was not enough information to determine a potential non-response bias for the remaining ten studies. Only one study presented information on the non-responders in order for the reader to determine issues of generalizability. Approximately 40% (8/19 studies) did not report psychometric properties nor referenced previous work demonstrating the reliability and validity of their weight intentions and weight loss strategy measures. Details pertaining to ethical approval or participant consent were not provided for the majority of studies (12/19). As studies reporting no sex difference, or a higher endorsement of weight loss strategies by males compared with females were published and reported in this systematic review, publication bias is suspected to be minimal.

**Table 2:** Quality assessment of included studies using the Quality of Cross-sectional Studies (AXIS) tool [37]

	Calderon et al. [18]	Childress et al. [24]	Davis & Lambert [25]	French et al. [46]	Kilpatrick et al. [19]	Koff & Rierdan [45]	Krowchuk et al. [23]	McVey et al. [20]	Page et al. [49]	Phelps et al. [40]	Rafiroiu et al. [43]	Serdula et al. [39]	Shisslak et al. [48]	Shisslak et al. [44]	Stevens et al. [41]	Story et al. [47]	Story et al. [21]	Yost et al. [38]	Zullig et al. [42]
1. Were the aims/objectives of the study clear?	Y	N	Y	Y	Y	Y	Y	Y	Y	Y	Y	Y	Y	Y	Y	Y	Y	Y	Y
2. Was the study design appropriate for the stated aim(s)?	Y	N/A^a^	Y	Y	Y	Y	Y	Y	Y	Y	Y	Y	Y	Y	Y	Y	Y	Y	Y
3. Was the sample size justified?	N	N	N	N	N	N	N	N	N	N	N	N	N	N	N	N	N	N	N
4. Was the target/reference population clearly defined? (Is it clear who the research was about?)	Y	Y	Y	Y	Y	Y	Y	Y	Y	Y	Y	Y	Y	Y	Y	Y	Y	Y	Y
5. Was the sample frame taken from an appropriate population base so that it closely represented the target/reference population under investigation?	Y	Y	Y	Y	Y	Y	Y	Y	Y	Y	Y	Y	Y	Y	Y	Y	Y	Y	Y
6. Was the selection process likely to select subjects/participants that were representative of the target/reference population under investigation?	Y	Y	N	N	Y	N	Y	Y	Y	N	Y	Y	Y	Y	Y	Y	Y	Y	Y
7. Were measures undertaken to address and categorize non-responders?	N	N	N	N	N	N	N	N	N	N	N	N	N	N	N	N	N	N	N
8. Were the weight intentions and weight strategies measured appropriate to the aims of the study?	Y	Y	N	Y	Y	Y	Y	Y	Y	Y	Y	Y	Y	Y	Y	Y	Y	Y	Y
9. Were the weight intentions and weight strategies measured correctly using instruments/measurements that had been trialed, piloted or published previously?	Y	Y	N	Y	Y	N	Y	N	Y	N	Y	Y	N	Y	N	N	Y	N	Y
10. Is it clear what was used to determined statistical significance and/or precision estimates? (eg, *p* values, CIs)	Y	Y	Y	Y	Y	N	Y	Y	N	N	Y	N/A	Y	N/A	Y	Y	N	Y	Y
11. Were the methods (including statistical methods) sufficiently described to enable them to be repeated?	Y	Y	Y	Y	Y	N	Y	Y	Y	N	Y	Y	N	Y	Y	Y	Y	Y	Y
12. Were the basic data adequately described?	Y	Y	Y	Y	Y	Y	Y	Y	Y	N	N	Y	Y	N	Y	Y	Y	Y	Y
13. Does the response rate raise concerns about non-response bias?	N	Y	N/A	N	N	N/A	N	Y	N/A	N/A	N/A	N	N/A	N/A	N	N/A	N/A	N/A	N
14. If appropriate, was information about non-responders described?	N	Y	N	N	N	N	N	N	N	N	N	N	N	N	N	N	N	N	N
15. Were the results internally consistent? (whether the numbers added up’, and ‘whether missing numbers were acknowledged or described’)	Y	Y	Y	Y	Y	Y	Y	Y	Y	Y	Y	Y	Y	Y	Y	Y	Y	Y	Y
16. Were the results for the analyses described in the methods, presented?	Y	Y	Y	Y	Y	N/A	Y	Y	Y	N/A	Y	Y	N/A	N/A	Y	Y	Y	Y	Y
17. Were the authors’ discussions and conclusions justified by the results?	Y	Y	Y	Y	Y	Y	Y	Y	Y	Y	Y	Y	Y	Y	Y	Y	Y	Y	Y
18. Were the limitations of the study discussed?	N	Y	Y	Y	Y	N	Y	Y	Y	N	Y	Y	Y	Y	Y	Y	Y	Y	Y
19. Were there any funding sources or conflicts of interest that may affect the authors’ interpretation of the results?	N/A	N/A	N/A	N	N/A	N	N/A	N	N/A	N/A	N	N/A	Y	Y	N	N	N	N	N/A
20. Was ethical approval or consent of participants attained?	N/A	Y	N/A	Y	N/A	Y	N/A	Y	N/A	N/A	Y	N/A	Y	N/A	N/A	N/A	N/A	N/A	Y

Note: ^a^N/A, information not available in article. For question 10, if the significance level for statistical tests was not provided, the item was graded as a ‘no’. For question 7, quality was assessed based on attempts to provide a rationale for response rates. For question 14, quality was assessed based on attempts to describe the non-responders relative to the responders. For question 16, if no analyses were proposed in the methods (i.e., prevalence and proportions proposed only), quality for presentation of results was deemed as N/A

For easier readability, overall prevalence information is presented first, followed by summary information organized by the sub-categories of weight status (separately for measured weight, and perceived weight), grade level, and race/ethnicity. A summary of all results presented is shown (Table 3).

**Table 3:** Prevalence statistics for weight loss intentions and strategies in youth by demographic characteristics and weight status

	Weight loss intention			Healthy			Unhealthy			Extreme		
	F^a^ (%^b^)	M^c^ (%)	Total	F (%)	M (%)	Total	F (%)	M (%)	Total	F (%)	M (%)	Total
Full sample	26–74	15–63	27–61	15–72	27–63	30–92	5–49	0–42	3–44	1–14	1–11	0–13
Measured Weight Status												
Underweight	--^d^	–	15–41	0–8	–	24–27	–	–	–	1–2	–	–
Normal-weight	–	–	38–52	10–18	–	36–73	–	–	–	1–2	–	–
Overweight/obese	–	–	70–82	27–33	–	47–75	–	–	–	1–3	–	–
Perceived weight status												
Underweight	–	–	2	30–38	–	–	0–2	–	–	0–7	–	–
Normal-weight	–	–	17	54–73	72	73	3–68	46	–	4–44	2–4	–
Overweight/obese	–	–	76	72–86	80	74	15–75	62	–	6–70	3–5	–
Grade Level												
Elementary	38–59	38–63	38–61	2–57	27–63	30–79	7–59	39–55	9–57	2–4	–	7
Middle	31–66	25–31	27–66	27–71	27–56	34–92	4–24	6–12	–	1–10	1–5	2–7
High school	43–47	15–37	30–60	–	–	30–60	17–49	10–42	9–41	1–15	1–11	2–13
Ethnicity												
Caucasian	47–58	16–25	32–46	55–75	26–78	34–76	4–73	3–57	8–69	2–11	3–5	1–8
African-American	30–48	10–27	21–39	26–61	28–73	30–64	12–69	2–54	10–66	3–9	2–7	1–7
Native-American	38–59	38–63	38–61	33–59	27–63	30–79	42–45	37–42	9–43	1–27	1–12	0–27
Hispanic	39	14	28–36	66	75	69	75	60	71	7–8	1–3	–
Asian-American	–	–	33	–	–	–	–	–	–	–	–	–

Note: ^a^F: Female; ^b^%: prevalence (range); ^c^Male;.^d^--: insufficient data. For specific references for each prevalence range, please refer to the text in the results section

#### Weight loss intentions

Of the 19 studies, the prevalence of those trying to lose weight was reported in 11 [18–21, 23–25, 39, 41–43], and ranged from 27 to 61%. In females, the prevalence ranged from 26 to 74% [18–21, 23–25, 38, 39, 41, 42, 44–46], while for males the prevalence ranged from 15 to 63% [18–21, 23–25, 39, 41, 42]. Of the studies that conducted independence tests examining sex differences in weight loss intentions, approximately two-thirds of these studies found that females endorsed trying to lose weight more often than males, while the remaining studies found no significant sex difference (Table 4).

**Table 4:** Significance test results for sex differences in weight loss intentions and strategies by demographic characteristics

			Studies finding sex difference	
		Studies finding no sex differences	Females higher endorsement	Males higher endorsement
Weight loss intentions				
Full sample		Davis & Lambert [25]^E,N^ Stevens et al. [41]^E,N^ Story et al. [21]^E,N^	Calderon et al. [18] Childress et al. [24]^M^ Kilpatrick et al. [19] Krowchuk et al. [23]^M^ McVey et al. [20]^M^	
Grade level				
	Elementary-school	Stevens et al. [41]^E,N^ Story et al. [21]^E,N^		
	Middle-school		Childress et al. [24]^M^ Krowchuk et al. [23]^M^ McVey et al. [20]^M^	
	High-school		Calderon et al. [18] Kilpatrick et al. [19]	
Type of strategy	Specific strategy			
Healthy				
	Exercise	McVey et al. [20]^M^ Story et al. [21]^E,N^	Childress et al. [24]^M^ Krowchuk et al. [23]^M^	Davis & Lambert [25]^E,N^ Kilpatrick et al. [19]
	Smaller portions	Calderon et al. [18]^H^	Davis & Lambert [25]^E,N^	
	Changed what/how ate	Story et al. [21]^E,N^		
Unhealthy				
	Fasting	Calderon et al. [18]^H^ Stevens et al. [41]^E,N^ Story et al. [21]^E,N^	Childress et al., 1993 [24]^M^	
	Skipping meals	Calderon et al. [18]^H^ Stevens et al. [41]^E,N^ Story et al. [21]^E,N^		McVey et al. [20]^M^
	Fad Diet	Calderon et al. [18]^H^		
Extreme				
	Diet pills	Calderon et al. [18]^H^ McVey et al. [20]^M^	Childress et al. [24]^M^ Krowchuk et al. [23]^M^ Story et al. [47]^N^	
	Diuretics	Childress et al. [24]^M^	Story et al. [47]^N^	
	Laxatives	Childress et al. [24]^M^		Story et al. [47]^N^
	Laxatives or diuretics			McVey et al. [20]^M^
	Vomiting	Childress et al. [24]^M^	Story et al. [47]^N^	McVey et al. [20]^M^
	Vomiting or laxatives		Krowchuk et al. [23]^M^	

Notes: No study conducted significance tests for sex differences within-race, nor within-weight status. Study characteristics are noted where study sample represents a single demographic characteristic, annotated as follows: ^E^Study sample composed of 100% elementary school aged participants; ^M^Study sample composed of 100% middle school aged participants; ^H^Study sample composed of 100% high school aged participants; ^N^Study sample composed of 100% Native-American participants

#### Weight loss strategy use

Among those studies reporting the total prevalence for strategy use (*n* = 12), 30–92% of participants used healthy [18–21, 23–25, 39, 41], 3–44% used unhealthy [18, 20, 21, 24, 25, 39, 41, 42], and 0–13% used extreme strategies to lose weight [18–20, 23, 24, 39, 42, 43, 47]. The proportion of females reporting the use of healthy practices was 15–72% [18–21, 23–25, 38, 39, 44, 45], while this prevalence in males ranged from 27 to 63% [18–21, 23–25, 39]. The prevalence of unhealthy strategies ranged from 5 to 49% in females [18, 20, 21, 24, 25, 39, 41, 42], and from 0 to 42% in males [18, 20, 21, 24, 25, 39, 41, 42]. Regarding the use of extreme strategies, 1–14% of females [18, 20, 23, 24, 39, 40, 42, 44, 46, 47] and 1–11% of males [18, 20, 23, 24, 39, 42, 47] reported using these to lose weight. Seven studies conducted significance tests examining sex differences in the use of healthy strategies, however findings were mixed (see Table 4).

### By weight status: Measured and perceived

Based on measured weight status, the total prevalence of overweight ranged from 22 to 42% [18, 19, 21, 25], from 21 to 43% in females [18, 21, 25, 38, 44], and 35–41% in males [18, 21, 25]. Based on perceived weight status, the prevalence of overweight ranged from 12 to 54% [18, 19, 21, 25], and ranged from 14 to 53% in females [18, 21, 25, 38, 44] and 9–58% in males [18, 21, 25].

#### Weight loss intentions

With regards to *measured* weight status, 70–82% of overweight/obese participants reported trying to lose weight [21, 25]. This was higher than the prevalence among normal-weight (38–52%) and underweight individuals (15–41%) [21, 25]. Regarding *perceived* weight status, 2% of underweight youth reported trying to lose weight, as did 17% of normal-weight and 76% of overweight/obese youth [39]. No study statistically examined sex differences in weight loss intentions among those in any weight status category.

#### Weight loss strategy use

Based on measured weight status, the total prevalence of using healthy weight loss strategies among underweight youth ranged from 24 to 27% [21]. Healthy strategies ranged from 36 to 73% [21], and 47–75% in normal-weight and overweight/obese youth, respectively [21]. Prevalence in males based on weight status was not available, but in females, the use of healthy strategies ranged from 0 to 8%, 10–18%, and 27–33% among underweight, normal-weight, overweight/obese females, respectively [44]. No study that reported measured weight status also reported prevalence of using unhealthy strategies. The use of extreme weight loss strategies was found to vary between 1 and 2% in underweight and normal-weight females, and varied between 1 and 3% among overweight/obese females [44]. Whether sex differences were significant between females and males were not reported.

Based on perceived weight status, the prevalence of using healthy weight loss strategies was 73% in normal-weight, and 74% in overweight/obese youth [39]. No study reported the use of healthy weight loss strategies among youth who perceived themselves to be underweight. Among females, 30–38%, 54–73% and 72–86% of underweight, normal-weight and overweight/obese youth reported using healthy weight loss strategies, respectively [39, 45]. Between 0 and 2% of females who perceived themselves as underweight reported using unhealthy strategies to lose weight, as did 3–68% of self-perceived normal-weight females, and 15–75% of self-perceived overweight/obese females [39, 45]. The prevalence of using extreme weight loss strategies was 0–7%, 4–44%, and 6–70% in self-perceived underweight, normal-weight, and overweight/obese females, respectively [39, 47]. Among males, 72% of those who perceived themselves as normal-weight and 80% of self-perceived overweight/obese males reported using healthy weight loss strategies [39]. For unhealthy weight loss strategies, the prevalence among males was 46% in normal-weight males and 62% in overweight/obese males [39]. Lastly, 2–4% of self-perceived normal-weight males and 3–5% of overweight/obese males reported using extreme weight loss strategies [39]. No study reported the use of weight loss strategies in males who perceived themselves to be underweight. Whether these proportions were significantly different between females and males were not reported in any study.

### By grade level

While difficult to determine the exact age students attend each grade level, generally speaking in the United States and Canada the school system is such that the ages are approximately 5–10 years of age during elementary school, 11–13 years of age during middle-school, and 14–18 years of age during high-school.

#### Weight loss intentions

The prevalence of overweight ranged from 12 to 42% in elementary school-aged children [21, 25, 41], 24–28% in middle school students, and from 25 to 54% in high school students [18, 39, 42]. The proportion of elementary school, middle school, and high school students reporting an intention to lose weight was 38–61%, 27–66% and 30–60%, respectively [18–21, 23–25, 39, 41–43].

The proportion of trying to lose weight among females and males in elementary school were similar (38–59% compared to 38–63%, respectively) [21, 25, 41, 48]. The proportion of trying to lose weight in middle school ranged from 31 to 66% among females and between 25 and 31% among males [20, 23, 24, 45, 48]. Among high school students, females consistently reported a higher prevalence of trying to lose weight than males, (43–74% compared to 15–37%, respectively) [18, 38, 39, 42]. There was no consistent sex difference in those trying to lose weight between elementary school males and females (Table 4) [21, 25, 41]. However, among both middle school [19, 24] and high school students [18, 39, 42], females were consistently found to report trying to lose weight more than males (Table 4).

#### Weight loss strategy use

Among elementary school students, 30–79% of students reported engaging in healthy weight loss strategies, while 9–57% reported the use of unhealthy strategies [21, 25, 41] and 7% reported using extreme strategies [43]. Among middle school students, 34–92% reported using healthy weight loss strategies, while 2–7% reported the use of extreme strategies [20, 23, 24]. Among high school students, 30–60% reported using healthy strategies for weight loss, while 9–41% of students reported using unhealthy methods and 2–13% reported using extreme strategies [18, 39, 42, 47].

The proportions observed for the use of healthy weight loss strategies in elementary and middle school females were 2–57% [21] and 27–71% [20, 21, 23–25, 45, 48], respectively. In elementary school males, prevalence of healthy strategies ranged from 27 to 63% [21, 25]. In middle school males, the prevalence of using healthy strategies ranged from 27 to 56% [20, 23, 24]. For unhealthy strategies, 7–59% of elementary school females, 4–24% of middle school females, and 17–49% of high school females reported their use [18, 20, 21, 24, 39, 42, 45, 48]. The prevalence of unhealthy strategies in elementary, middle, and high school males was 39–55% and 6–12% and 10–42%, respectively [18, 20, 21, 24, 39, 42]. Regarding the use of extreme weight loss strategies, one study [48] found that 2–4% of elementary school females reported using these, while others reported larger ranges of prevalence for extreme strategies among middle school females and high school females (1–10% and 1–15%, respectively) [18, 20, 23, 24, 39, 40, 42, 47, 48]. The proportion of males using extreme weight loss methods was 1–5% and 1–11%, respectively [18, 47] in middle school and high school aged youth.

Three studies of elementary school students [21, 25, 41], three studies of middle school students [20, 23, 24], and one study of high school students [18] reported sex differences in the use of particular weight loss strategies (included in aggregate in Table 4, see annotations).

### By race/ethnicity

Of note, the terms used here to describe race/ethnicity have been chosen to represent a set of interchangeable terms provided in the original articles, and are grouped as follows: Study participants referred to in the original articles as Caucasian, White or non-Hispanic white are referred to here as “Caucasian.” Those referred to as African-American, African-Canadian, or Black are referred to here as “African-American.” Those referred to as Hispanic, Mexican-American, or Puerto Rican are referred to here as “Hispanic”. Study participants referred to as Native-American, American-Indian, or Alaska Native in the original articles are referred to here as “Native-American.” Those referred to as Asian-American, Asian, Pacific Islander, or South Asian are referred to here as “Asian-American”.

#### Weight loss intentions

The prevalence of trying to lose weight in Caucasian youth ranged from 32 to 46% [19, 24, 39, 42], from 21 to 39% in African-American youth [19, 24, 39, 42], from 38 to 61% in Native-American youth [19, 21, 25, 41], from 28 to 36% in youth of Hispanic origin [19, 39], and 33% in those identifying as Asian-American [19].

The prevalence of trying to lose weight by race/ethnicity based on sex was: 47–58% in Caucasian females compared to 16–25% Caucasian males; 30–48% in African-American females compared to 10–27% of African-American males [39, 42]; 39% in Hispanic females compared to 14% in Hispanic males [39], 38–59% in Native-American females and 38–63% in Native-American males [21, 25, 41]. This information was not available for Asian-American males or females. No study conducted significance tests examining sex differences in weight loss intentions within-race/ethnicity.

#### Weight loss strategy use

The prevalence of using healthy weight loss strategies in Caucasian youth ranged from 34 to 76% [24, 39]. Among Caucasian females, this prevalence ranged from 55 to 75%, and from 26 to 78% in Caucasian males [24, 39, 45, 49]. Among African-American youth, 30–64% used healthy strategies to lose weight [24, 39], and 26–61% of females and 28–73% of males participated in healthy weight loss strategies [24, 39, 49]. In Native-American youth, 30–79% used healthy weight loss strategies [21, 25, 41]. Thirty-three to 59% of Native-American females and 27–63% of males reported using healthy strategies to lose weight [21, 25, 41]. Sixty-nine percent of Hispanic youth used healthy strategies to lose weight [39]. Of these youth, 66% of females and 75% of Hispanic males reported using healthy strategies to lose weight [39].

The use of unhealthy weight loss strategies was practiced by 8–69% of Caucasian youth [24, 39, 42], ranging from 4 to 73% in females and 3–57% in males [39, 42, 45, 49]. In African-American youth, the total prevalence of using unhealthy weight loss strategies ranged from 10 to 66% [22, 46, 49]; from 12 to 69% in females and from 2 to 54% in males [39, 42, 49]. Approximately 9–43% of Native-American youth reported using unhealthy strategies [21, 25, 41], ranging from 42 to 45% (females) and 37–42% (males) [21]. In Hispanic youth, 71% reported using unhealthy strategies; (75% of females and 60% of males) [39].

The prevalence of using extreme weight loss strategies ranged from 1 to 8% among Caucasian youth [24, 42]. For Caucasian females, this ranged from 2 to 11%, and from 3 to 5% in males [39, 42, 49]. In African-American youth, 1–7% reported using extreme weight loss strategies [24, 42]. In African-American females, this ranged from 3 to 9%, and from 2 to 7% in African-American males [39, 42, 49]. The total prevalence for using extreme strategies in Native-American youth ranged from 0 to 27% [24, 47], and from 1 to 27% in females and 1–12% in males [21, 41]. In Hispanic females, the prevalence of using extreme strategies ranged from 7 to 8% and from 1 to 3% in males [39]. While no study statistically examined the presence of sex differences within-race/ethnicity, three studies whose participants were 100% Native-American youth reported sex differences in the use of particular strategies, however results were mixed (Table 4, see annotations).

## Discussion

The objective of the current paper was to review the existing literature on weight loss intentions and weight loss strategy use among youth, and to compile prevalence estimates of these based on demographic characteristics and weight status. Of particular interest was the question of whether patterns within these characteristics differed by sex.

### Weight loss intentions

Our results demonstrate that up to two-thirds of youth report trying to lose weight. The prevalence of trying to lose weight reached 74% in females and 63% in males, however results from statistical comparisons show mixed findings, with some studies reporting no sex difference in weight loss intention, and some reporting higher endorsement among females. Mixed findings may be observed due to differences in demographic characteristics, such as age and race/ethnicity. For instance, several studies report no sex difference in weight loss intentions among elementary school children, while studies conducted among middle and high school students consistently report that females are more likely than males of the same age to be trying to lose weight. Indeed, research has demonstrated that children as young as 8 years old express concerns about their weight and body shape [50], and that these concerns, as well as actions taken to control body weight, increase substantially as youth move from elementary to middle school [48]. This increase in attention to body weight in young females is likely due to a combination of factors, including onset of menarche, dating, and increased peer pressure [48], as well as increased exposure to societal ideals of beauty through television and social media during childhood and emerging adolescence [51]. While the overall finding that young females are more likely to be trying to lose weight compared to males is consistent with findings among adults [52], the reasons for wanting to lose weight are likely qualitatively different. Indeed, while both adults and adolescents cite self-esteem as an important factor in wanting to lose weight, research suggests that children are more motivated by a desired appearance when trying to lose weight compared to adults, who cite health as a primary concern [53, 54].

In addition, parent/peer influences have been shown to shape weight loss intentions [55–58]. This is likely a confluence of behaviour modeling, social support, and parental/peer pressure. Although research is limited, it does appear that the importance of these influences varies by age and sex. Further research into the influence of these social networks is needed, as the importance of these social networks on body image, self-esteem, and weight intention may be critical to preventing maladaptive weight loss habits. In particular, as it appears that once young females reach middle and high school their intentions of maintaining a particular body shape are already exaggerated compared to boys, understanding the influence of these social networks on younger females may be especially critical from a public health perspective. Thus, public health efforts should consider targeting elementary school children to encourage the development of a healthy body image and lifestyle. Given that sex differences in weight loss intentions persist into adulthood [52], a more comprehensive evaluation of which concerns and attitudes about weight loss persist from childhood into adulthood is certainly necessary.

With regards to race/ethnicity, Caucasian, African-American, and Hispanic females appear to report consistently higher prevalence of trying to lose weight compared to males in the same group, while rates were similar among Native-American females and males. Of note, Native-American males demonstrated a higher pattern of endorsement of weight loss intentions compared to all other ethnicities examined. Such observations are important given that previous research indicates important distinctions between race/ethnicity with regard to weight perceptions and management, largely due to cultural differences. For instance, one study suggests that body weight concerns in young females across cultures are influenced by media messaging [59], while another implies that consumption of media targeted to African-Americans is unrelated to body weight concerns [60]. A better understanding of factors associated with race/ethnicity and weight intentions is warranted, especially given the disparity in the prevalence of overweight among African-American, Native-American, and Caucasian youth in the United States [61] and Canada [62].

Unsurprisingly, it was observed that a larger proportion of overweight/obese participants reported trying to lose weight compared to normal-weight and underweight individuals (based on measured weight status), but sex differences were not reported. The lack of data on sex differences across these categories represents a major limitation as research shows that young females are more likely to perceive themselves as overweight compared to young males [19]. Further, the literature suggests that perceived weight status is a stronger predictor of weight intentions and strategies than measured weight status. For instance, preliminary evidence among adults suggests that perceived weight status may fully mediate the association between measured weight and weight intentions [63, 64] but differs by sex and race/ethnicity. Whether this mediation occurs across sex and race/ethnicity among adolescents should be further evaluated.

### Weight loss strategies

The proportion of youth using unhealthy or extreme strategies is as high as 44 and 13%, respectively. Overall, a similar proportion of males and females endorsed the use of each category of weight loss strategies across studies. This similarity is important to note given the tendency for the literature to focus on females when examining weight loss behaviours. This phenomenon is reflected in the studies included in this review, of which 32% had all-female samples, and no studies examining all-male samples. While trends do suggest that females are more concerned with weight loss while males are more concerned with increasing muscle [65], an overemphasis on young females may obscure the rate of risky behaviours engaged in by young males who are trying to lose weight, including the use of unhealthy and extreme strategies.

Still, given the differences in motivating factors, young females may require special attention with regards to education about weight loss behaviours. As noted, research suggests that appearance is a primary motivator for young females to lose weight, rather than health. Thus, additional efforts are needed to promote healthy body shape and body image in young females, to supplement the promotion of health. Doing so is likely to decrease the frequency with which youth are engaging in these dangerous behaviours. Indeed, research suggests that the use of unhealthy and extreme weight loss strategies is linked to other risky behaviours (e.g., using indoor tanning beds, using drugs) [66], some of which are likely also motivated by appearance concerns.

Results of this review demonstrate the prevalence of using extreme weight loss strategies was as high as 27% in Native-American young females - the highest of any race/ethnicity examined for that category. Four studies included in the review examined samples of Native-Americans exclusively, but demonstrated no clear pattern regarding the preference to use specific strategies by sex. The use of unhealthy strategies was observed to be as high as 69% in young African-American females, 73% in young Caucasian females, and 75% in young Hispanic females. Given the observational nature of this study, however, no conclusions can be drawn regarding risk for females in these groups at this time, and further research is needed.

Surprisingly, very little data was available regarding the use of particular weight loss strategies across weight status categories based on measured weight status. Of note is the contrast among endorsement rates for the use of extreme strategies between measured and perceived weight status, where 1–3% of females across weight categories endorsed their use based on measured weight status, but that up to 44 and 70% of normal weight and overweight/obese females endorse the use of extreme strategies based on perceived size. As noted above, this misperception of weight in youth appears to play a role in the weight loss strategies favoured by youth, and may lead to the use of maladaptive behaviours. Again, while sex differences for the use of particular weight loss strategies were not examined in any study, it appears as though young females are using extreme strategies to a much greater extent than male counterparts in the same weight category, a difference that does not appear across the use of healthy or unhealthy strategies. Given the significant lack of information in this area, further research is urgently needed to examine how weight status, body image perceptions, and sex are related to weight control behaviours.

### Limitations

This systematic review focused on Canadian and American youth, however variability in study methodology and quality precluded the carrying out of a meta-analysis. Insights into sex differences across demographic characteristics and weight status were thus weakened by the limited literature and more studies focusing on weight loss intentions and strategies specifically are needed. Further, results from this review cannot be generalized beyond the United States and Canada.

Study quality and rigour was decreased primarily due to a lack of methodological information (such as a sample size calculation, psychometric properties of measures, and response rates). Although some of these details may be more pertinent to cohort studies, the quality assessment tool used was specifically designed to evaluate cross-sectional studies. Studies were excluded if they did not provide sufficient data necessary for inclusion in this systematic review (such as weight loss strategies which were too broadly defined). Although it is possible that these studies did not collect greater detailed information on weight intentions and weight loss strategy use, it is also possible that several categories were collapsed due to insufficient numbers. This may impact the lower bounds of our estimates such that they are overestimated.

One major limitation of the existing literature is our observation that no study assessing weight loss intentions and strategy use examined the use of multiple strategies in tandem with one another. This is an important concern, as while the high rates of endorsement for the use of healthy weight loss strategies is encouraging, it is unclear the extent to which these are being used alongside more deleterious methods. Another important limitation to note is that no study that met criteria for inclusion in this review reported prevalence information based on socio-economic characteristics. Given the well documented relationship among race/ethnicity, socio-economic status, and health [67, 68], researchers need to report basic data for such characteristics when examining health and weight loss behaviours, and should consider these factors in their analyses.

### Implications

Several important implications from this systematic review are apparent. As considerable methodological variation in study measures were found, further research on harmonizing these measures are needed in order for meta-analyses to be conducted and more reliable conclusions to be drawn. For instance, weight loss strategies were commonly described in very broad terms (e.g., “dieting”), and effort in creating stricter guidelines with regards to measurement methodology for research on weight loss behaviours is needed. Better defining strategies may be particularly important as research indicates that dietary changes are reportedly stronger contributors to weight loss than exercise [69, 70]. Similarly, there was considerable variability in the measurement of weight loss intentions, which is commonly measured as either ‘current’ intentions, or lifetime intentions. Thus there is a concern for recall bias, particularly from the studies which use ‘lifetime’ intentions.

Further, weight loss and weight maintenance intentions are commonly grouped together into a single survey question when examining weight change strategies (e.g., which of the following strategies did you use to “to lose weight or keep from gaining weight”). Very few studies reported weight loss strategy use separately from weight maintenance, resulting in the relatively small sample of studies included in this review. This is an important distinction given that weight loss and weight maintenance are cognitively different goals, and lead to differences in the use of particular weight change strategies [32, 71]. Thus, grouping them together may obscure these important differences.

The literature is limited by an over-emphasis on cross-sectional studies, and the longitudinal implications of using healthy, unhealthy or extreme strategies remain unclear. In particular, it is unknown whether the age at which youth first attempt weight loss is associated with the types of strategies used. Additional research is needed to refine our understanding of how weight loss behaviours are developed and maintained in youth.

### Conclusion

In this systematic review, several patterns emerged regarding the prevalence of weight loss intentions and strategy use between young females and males across demographic groups and by weight status. However, insufficient data based on race/ethnicity and weight status limit our abilities to conclude whether sex differences based on these characteristics exist, and further research is needed. The development of standardized measures and guidelines for research methodology related to weight loss intentions and strategies in youth is also warranted to reliably assess trends in future research.

## Additional file

Additional file 1: Electronic Search Strategy. Full electronic search strategy conducted in PubMED, Web of Science, and PsycInfo. (DOCX 13.6 kb)

## Abbreviations

BMI: Body mass index

## References

[1] Demissie Z, Lowry R, Eaton DK, Nihiser AJ (2015). Trends in weight management goals and behaviors among 9th–12th grade students: United States, 1999–2009. Matern Child Health J.

[2] Lowry R, Galuska DA, Fulton JE, Wechsler H, Kann L (2002). Weight management goals and practices among US high school students: associations with physical activity, diet, and smoking. J Adolesc Health.

[3] Gow ML, Baur LA, Ho M, Chisholm K, Noakes M, Cowell CT (2016). Can early weight loss, eating behaviors and socioeconomic factors predict successful weight loss at 12- and 24-months in adolescents with obesity and insulin resistance participating in a randomised controlled trial?. Int J Behav Nutr Phys Act..

[4] Boutelle KN, Hannan PJ, Neumark-Sztainer D, Himes JH (2007). Identification and correlates of weight loss in adolescents in a national sample. Obesity..

[5] Alm ME, Neumark-Sztainer D, Story M, Boutelle KN (2009). Self-weighing and weight control behaviors among adolescents with a history of overweight. J Adolesc Health.

[6] Walker SE, Smolkin ME, O’Leary MLL, Cluett SB, Norwood VF, DeBoer MD (2012). Predictors of retention and BMI loss or stabilization in obese youth enrolled in a weight loss intervention. Obes Res Clin Pract..

[7] Barlow SE (2007). Expert committee recommendations regarding the prevention, assessment, and treatment of child and adolescent overweight and obesity: summary report. Pediatrics.

[8] Field AE, Haines J, Rosner B, Willett WC (2010). Weight-control behaviors and subsequent weight change among adolescents and young adult females. Am J Clin Nutr.

[9] Neumark-Sztainer D, Wall M, Story M, Standish AR (2012). Dieting and unhealthy weight control behaviors during adolescence: associations with 10-year changes in body mass index. J Adolesc Health.

[10] Boutelle KN, Libbey H, Neumark-Sztainer D, Story M (2009). Weight control strategies of overweight adolescents who successfully lost weight. J Am Diet Assoc.

[11] Gallant AR, Perusse-Lachance E, Provencher V, Begin C, Drapeau V (2013). Characteristics of individuals who report present and past weight loss behaviours: results from a Canadian university community. Eat Weight Disord-Stud Anorex Bulim Obes.

[12] Gusella J, Goodwin J, van Roosmalen E (2008). “I want to lose weight”: early risk for disordered eating?. Paediatr Child Health.

[13] Stephen EM, Rose JS, Kenney L, Rosselli-Navarra F, Weissman RS (2014). Prevalence and correlates of unhealthy weight control behaviors: findings from the national longitudinal study of adolescent health. J Eat Disord.

[14] Forney KJ, Buchman-Schmitt JM, Keel PK, Frank GKW (2016). The medical complications associated with purging. Int J Eat Disord.

[15] Field AE, Colditz GA, Peterson KE (1997). Racial/ethnic and gender differences in concern with weight and in bulimic behaviors among adolescents. Obes Res.

[16] Boutelle K, Neumark-Sztainer D, Story M, Resnick M (2002). Weight control behaviors among obese, overweight, and nonoverweight adolescents. J Pediatr Psychol.

[17] Eisenberg ME, Neumark-Sztainer D, Story M, Perry C (2005). The role of social norms and friends’ influences on unhealthy weight-control behaviors among adolescent girls. Soc Sci Med.

[18] Calderon LL, Yu CK, Jambazian P (2004). Dieting practices in high school students. J Am Diet Assoc.

[19] Kilpatrick M, Ohannessian C, Bartholomew JB (1999). Adolescent weight management and perceptions: an analysis of the National Longitudinal Study of adolescent health. J Sch Health.

[20] McVey G, Tweed S, Blackmore E (2005). Correlates of weight loss and muscle-gaining behavior in 10-to 14-year-old males and females. Prev Med.

[21] Story M, Stevens J, Evans M (2001). Weight loss attempts and attitudes toward body size, eating, and physical activity in American Indian children: relationship to weight status and gender. Obes Res.

[22] Wu T-Y, Rose SE, Bancroft JM (2006). Gender differences in health risk behaviors and physical activity among middle school students. J Sch Nurs.

[23] Krowchuk DP, Kreiter SR, Woods CR, Sinal SH, DuRant RH (1998). Problem dieting behaviors among young adolescents. Arch Pediatr Adolesc Med..

[24] Childress AC, Brewerton TD, Hodges EL, Jarrell MP (1993). The kids’ eating disorders survey (KEDS): a study of middle school students. J Am Acad Child Adolesc Psychiatry.

[25] Davis SM, Lambert LC (2000). Body image and weight concerns among southwestern American Indian preadolescent schoolchildren. Ethn Dis.

[26] Offord DR, Kraemer HC, Kazdin AE, Jensen PS, Harrington R (1998). Lowering the burden of suffering from child psychiatric disorder: trade-offs among clinical, targeted, and universal interventions. J Am Acad Child Adolesc Psychiatry.

[27] Solomons NW (2005). Programme and policy issues related to promoting positive early nutritional influences to prevent obesity, diabetes and cardiovascular disease in later life: a developing countries view. Matern Child Nutr.

[28] Poobalan AS, Aucott LS, Precious E, Crombie IK, Smith WCS (2010). Weight loss interventions in young people (18 to 25 year olds): a systematic review. Obes Rev.

[29] Booth HP, Prevost TA, Wright AJ, Gulliford MC (2014). Effectiveness of behavioural weight loss interventions delivered in a primary care setting: a systematic review and meta-analysis. Fam Pract.

[30] Thomason DL, Lukkahatai N, Kawi J, Connelly K, Inouye J (2016). A systematic review of adolescent self-management and weight loss. J Pediatr Health Care.

[31] Mühlig Y, Wabitsch M, Moss A, Hebebrand J (2014). Weight loss in children and adolescents. Dtsch Ärztebl Int.

[32] Sciamanna CN, Kiernan M, Rolls BJ (2011). Practices associated with weight loss versus weight-loss maintenance: results of a national survey. Am J Prev Med.

[33] López-Guimerà G, Neumark-Sztainer D, Hannan P, Fauquet J, Loth K, Sánchez-Carracedo D (2013). Unhealthy weight-control behaviours, dieting and weight status: a cross-cultural comparison between north American and Spanish adolescents. Eur Eat Disord Rev.

[34] Clarivate Analytics. Endnote X7 [Computer software]. Philadelphia, PA: Thompson Reuters; 2015.

[35] Neumark-Sztainer D, Wall M, Eisenberg ME, Story M, Hannan PJ (2006). Overweight status and weight control behaviors in adolescents: longitudinal and secular trends from 1999 to 2004. Prev Med.

[36] Neumark-Sztainer D, Rock CL, Thornquist MD, Cheskin LJ, Neuhouser ML, Barnett MJ (2000). Weight-control behaviors among adults and adolescents: associations with dietary intake. Prev Med.

[37] Downes MJ, Brennan ML, Williams HC, Dean RS (2016). Development of a critical appraisal tool to assess the quality of cross-sectional studies (AXIS). BMJ Open.

[38] Yost J, Krainovich-Miller B, Budin W, Norman R (2010). Assessing weight perception accuracy to promote weight loss among U.S. female adolescents: a secondary analysis. BMC Public Health.

[39] Serdula MK, Collins ME, Williamson DF, Anda RF, Pamuk E, Byers TE (1993). Weight control practices of U.S. adolescents and adults. Ann Intern Med.

[40] Phelps L (1993). RonRizzo, frank G Johnston LisaMain, Colleen M. prevalence of self-induced vomiting and laxative/medication abuse among female adolescents: a longitudinal study. Int J Eat Disord..

[41] Stevens J, Story M, Becenti A (1999). Weight-related attitudes and behaviors in fourth grade American Indian children. Obes Res.

[42] Zullig K, Ubbes VA, Pyle J, Valois RF (2006). Self-reported weight perceptions, dieting behavior, and breakfast eating among high school adolescents. J Sch Health..

[43] Rafiroiu AC, Anderson EP, Sargent RG, Parra-Medina D, Jackson KL, Thompson S (2000). Factors associated with diet and dieting behavior of elementary school children. J Child Nutr Manag.

[44] Shisslak CM, Mays MZ, Crago M, Jirsak JK, Taitano K, Cagno C (2006). Eating and weight control behaviors among middle school girls in relationship to body weight and ethnicity. J Adolesc Health.

[45] Koff E, Rierdan J (1991). Perceptions of weight and attitudes toward eating in early adolescent girls. J Adolesc Health.

[46] French SA, Perry CL, Leon GR, Fulkerson JA (1995). Dieting behaviors and weight change history in female adolescents. Health Psychol.

[47] Story M, Hauck FR, Broussard BA, White LL, Resnick MD, Blum RW (1994). Weight perceptions and weight control practices in American Indian and Alaska native adolescents. Arch Pediatr Adolesc Med.

[48] Shisslak CM, Crago M, McKnight KM, Estes LS, Gray N, Parnaby OG (1998). Potential risk factors associated with weight control behaviors in elementary and middle school girls. J Psychosom Res.

[49] Page RM, Allen O, Moore L, Hewitt C (1993). Weight-related concerns and practices of male and female adolescent cigarette smokers and nonsmokers. J Health Educ.

[50] McCabe MP, Ricciardelli LA (2003). Body image and strategies to lose weight and increase muscle among boys and girls. Health Psychol.

[51] Morris AM, Katzman DK (2003). The impact of the media on eating disorders in children and adolescents. Paediatr Child Health.

[52] Tsai SA, Lv N, Xiao L, Ma J (2016). Gender differences in weight-related attitudes and behaviors among overweight and obese adults in the United States. Am J Mens Health.

[53] Murtagh J, Dixey R, Rudolf M (2006). A qualitative investigation into the levers and barriers to weight loss in children: opinions of obese children. Arch Dis Child.

[54] Brink PJ, Ferguson K (1998). The decision to lose weight. West J Nurs Res.

[55] Brown CL, Skelton JA, Perrin EM, Skinner AC (2016). Behaviors and motivations for weight loss in children and adolescents. Obes Silver Spring Md.

[56] Farrow CV, Haycraft E, Blissett JM (2015). Teaching our children when to eat: how parental feeding practices inform the development of emotional eating--a longitudinal experimental design. Am J Clin Nutr.

[57] Vander Wal JS (2012). The relationship between body mass index and unhealthy weight control behaviors among adolescents: the role of family and peer social support. Econ Hum Biol.

[58] Balantekin KN, Savage JS, Marini ME, Birch LL (2014). Parental encouragement of dieting promotes daughters’ early dieting. Appetite.

[59] Madden D, Breny JM (2016). “How should I be?” a photovoice exploration into body image messaging for young women across ethnicities and cultures. Health Promot Pract.

[60] Adams-Bass VN, Stevenson HC, Kotzin DS (2014). Measuring the meaning of black media stereotypes and their relationship to the racial identity, black history knowledge, and racial socialization of African American youth. J Black Stud.

[61] Childhood obesity facts: overweight & obesity. https://www.cdc.gov/obesity/data/childhood.html. Accessed 9 May 2017.

[62] Overweight Canadian children and adolescents. http://www.statcan.gc.ca/pub/82-620-m/2005001/article/child-enfant/8061-eng.htm. Accessed 9 May 2017.

[63] Fan M, Jin Y (2015). The effects of weight perception on adolescents’ weight-loss intentions and behaviors: evidence from the Youth Risk Behavior Surveillance Survey. Int J Environ Res Public Health..

[64] Assari Shervin, Lankarani MaryamMoghani (2015). Mediating effect of perceived overweight on the association between actual obesity and intention for weight control; role of race, ethnicity, and gender. International Journal of Preventive Medicine.

[65] McCabe MP, Busija L, Fuller-Tyszkiewicz M, Ricciardelli L, Mellor D, Mussap A (2015). Sociocultural influences on strategies to lose weight, gain weight, and increase muscles among ten cultural groups. Body Image.

[66] O’Riordan DL, Field AE, Geller AC (2006). Frequent tanning bed use, weight concerns, and other health risk behaviors in adolescent females (United States). Cancer Causes Control.

[67] Fradkin C, Wallander JL, Elliott MN, Tortolero S, Cuccaro P, Schuster MA (2015). Associations between socioeconomic status and obesity in diverse, young adolescents: variation across race/ethnicity and gender. Health Psychol.

[68] Wang Y, Beydoun MA (2007). The obesity epidemic in the United States - gender, age, socioeconomic, racial/ethnic, and geographic characteristics: a systematic review and meta-regression analysis. Epidemiol Rev.

[69] Schwingshackl L, Dias S, Hoffmann G (2014). Impact of long-term lifestyle programmes on weight loss and cardiovascular risk factors in overweight/obese participants: a systematic review and network meta-analysis. Syst Rev.

[70] Thomas DM, Bouchard C, Church T (2012). Why do individuals not lose more weight from an exercise intervention at a defined dose? An energy balance analysis. Obes Rev Off J Int Assoc Study Obes.

[71] McGuire MT, Wing RR, Klem ML, Seagle HM, Hill JO (1998). Long-term maintenance of weight loss: do people who lose weight through various weight loss methods use different behaviors to maintain their weight?. Int J Obes Relat Metab Disord.

