# Supplementary material for: Do sex differences in reported weight loss intentions and behaviours persist across demographic characteristics and weight status in youth? A systematic review
Source: BMC Public Health. 2018 Dec 4;18:1343. doi: 10.1186/s12889-018-6179-x (PMC6280345; doi:10.1186/s12889-018-6179-x)
Supplement: Supplementary file 1 — Electronic Search Strategy. Full electronic search strategy conducted in PubMED, Web of Science, and PsycInfo. (DOCX 13.6 kb) [file 12889_2018_6179_MOESM1_ESM.docx]

**Additional file 1**

**Electronic Search Strategy**

#1 youth*

#2 teen*

#3 adolescen*

#4 “high school”

#5 “school age*”

#6 “middle school”

#7 child*

#8 “weight loss”

#9 “lose weight”

#10 “lost weight”

#11 “weight lost”

#12 strateg*

#13 intent*

#14 goal*

#15 try*

(#1 OR #2 OR #3 OR #4 OR #5 OR #6 OR #7)

AND

(#8 OR #9 OR #10 OR #11)

AND

(#12 OR #13 OR #14 OR #15)
